# Metastatic Breast Cancer and Pre-Diagnostic Blood Gene Expression Profiles—The Norwegian Women and Cancer (NOWAC) Post-Genome Cohort

**DOI:** 10.3389/fonc.2020.575461

**Published:** 2020-10-15

**Authors:** Einar Holsbø, Karina Standahl Olsen

**Affiliations:** ^1^Department of Computer Science, UiT The Arctic University of Norway, Tromsø, Norway; ^2^Department of Community Medicine, UiT The Arctic University of Norway, Tromsø, Norway

**Keywords:** breast cancer, metastasis, transcriptomics, blood, immune system, Bayesian data analysis, causal diagrams

## Abstract

Breast cancer patients with metastatic disease have a higher incidence of deaths from breast cancer than patients with early-stage cancers. Recent findings suggest that there are differences in immune cell function between metastatic and non-metastatic cases, even years before diagnosis. We have analyzed whole blood gene expression by Illumina bead chips in blood samples taken using the PAXgene blood collection system up to two years before diagnosis. The final study sample included 197 breast cancer cases and 197 age-matched controls. We defined a causal directed acyclic graph to guide a Bayesian data analysis to estimate the risk of metastasis associated with the expression of all genes and with relevant sets of genes. We ranked genes and gene sets according to the sign probability for excess risk. Among the screening detected cancers, 82% were without metastasis, compared to 53% of between-screening detected cancers. Among the highest ranking genes and gene sets associated with metastasis risk, we identified plasmacytiod dentritic cell function, the SLC22 family of transporters, and glutamine metabolism as potential links between the immune system and metastasis. We conclude that there may be potentially wide-reaching differences in blood gene expression profiles between metastatic and non-metastatic breast cancer cases up to two years before diagnosis, which warrants future study.

## Introduction

In recent decades, survival of breast cancer has increased substantially (1). However, among breast cancer patients, the proportion of deaths due to breast cancer increases with advanced tumor stage, particularly for metastatic cancer (2). Improving our understanding of metastatic disease may lead to better diagnosis and increased survival.

The host immune response plays an important role in modulating the progression of cancer, including the progression of metastasis (3). The fate of a disseminated cancer cell depends on its interactions with the immune cells it encounters during its transit through the circulatory system. Its fate also depends on escaping from clearance by the immune system (4). A study on node-positive (metastatic) and node-negative (non-metastatic) breast cancer patients showed different mRNA gene expression patterns, both in tumors and lymph nodes, but also in the peripheral blood (5). In the blood cells of non-metastatic patients, gene expression patterns related to lymphocyte activation and B-cells were up-regulated, indicating a systemic down-regulation of immune function in patients with metastasis (5).

The diagnostic potential of blood gene expression profiles for breast cancer has been investigated in blood samples taken at the time of diagnosis (6). But so far only diagnostic gene expression tests based on tumor tissue have reached clinical use (7). Still, previous findings from the NOWAC Post-genome cohort suggest that blood gene expression profiles differ between future breast cancer cases and healthy controls up to 8 years before diagnosis, stratifying on cancer stage and mode of detection (8, 9). Routine mammography screening in Norway is offered every two years to women over the age of 50. Mammography-detected cancers are found at an earlier stage of the carcinogenic process compared to clinically detected cancers (10). Interval cancers, i.e., those that are detected in the interval between screenings, are often of a more aggressive type, as they arise and are clinically detected less than two years after a screening mammogram (11).

In this study we used whole-genome gene expression data from 197 breast cancer cases and age-matched controls from the Norwegian Women and Cancer (NOWAC) Post-genome cohort. Our aim was to investigate the potential differences in blood gene expression profiles between patients with metastasized cancer and patients with non-metastasized cancer. This is an exploratory analysis to uncover promising avenues for future research. Hence, we do not focus on hypothesis testing and control of the error rates associated with these procedures. Instead, we apply Bayesian modeling to shrink estimates toward reasonable ranges.

## Materials and Methods

The NOWAC study is a nationally representative, prospective questionnaire-based cohort of approximately 170 000 middle-aged women (12). Among NOWAC participants, approximately 50 000 women born in 1943-1957, were randomly selected and invited to participate in the NOWAC Post-genome cohort (13). During the years 2003-2006, these women provided blood samples and additional questionnaires on lifestyle and reproductive factors at the time of blood sampling. The blood samples were collected using the PAXgene Blood RNA system (Preanalytix/Qiagen, Hilden, Germany), which preserves the RNA profile of the blood sample for future transcriptomic analysis.

The Cancer Registry of Norway provided information on mammography screening attendance and clinical information on cancer diagnoses. The most recent cancer registry update for the present study is from 2017. We defined breast cancer cases with a positive lymph node status as metastatic cases. Breast cancer subtypes were defined in accordance with the consensus (14) on clinical and molecular classification of breast cancer tumors (15, 16). This is based on hormone receptor status (estrogen receptor: ER, and progesterone receptor: PR) and human epidermal growth factor receptor 2 (Her2). There was some missing information on receptor and/or HER2 status: ER: 3 missing, PR: 3 missing, and Her2: 19 missing. These cases were defined as subtype unknown. Follow-up time was defined as the number of days between the date of blood sample donation and the date of diagnosis.

For our study, we started out with 231 women who were diagnosed with breast cancer at most two years after providing a blood sample. We drew age-matched, healthy controls from the NOWAC Post-genome cohort. Due to missing data on height, weight, or HRT use, we excluded seven cases and their corresponding controls. We also excluded nine case/control pairs due to missing information on screening attendance, and three due to missing metastasis status. Finally, we excluded 15 case/control pairs due to missing gene expression data for the control. This left a final study sample for data analysis of 197 breast cancer cases and 197 age-matched controls. Since we compared cases with metastasis to cases without metastasis, the controls served merely as a normalization of expression levels. This is primarily useful to mitigate batch effects.

We performed all data processing and analysis in R, using the Bioconductor and rstan packages (https://www.r-project.org/). The code we produced for this project is available online at https://github.com/uit-hdl/holsbo_olsen_2020.

### Laboratory Analyses and Data Pre-Processing

The Illumina-certified Genomics Core Facility at the Norwegian University of Science and Technology processed the blood samples. We kept each case-control pair together throughout the lab procedures to minimize technical variability, since the pairs are always processed at the same time in the same batches. Total RNA was isolated in accordance with the manufacturer’s protocol (PAXgene Blood miRNA isolation Kit). RNA purity was assessed by NanoDrop ND 8000 spectrophotometer (ThermoFisher Scientific, Wilmington, DE, USA), and RNA integrity by Bioanalyzer capillary electrophoresis (Agilent Technologies, Palo Alto, CA, USA). mRNA was amplified and labeled using the Illumina TotalPrepT-96 RNA Amplification Kit (Ambion Inc., Austin, TX, USA), and hybridized to Illumina HumanWG-6 v.3 Expression BeadChip microarrays (Illumina, Inc. San Diego, CA, USA). The raw microarray images were processed in Illumina GenomeStudio.

We performed preprocessing of the raw microarray data according to the NOWAC standard procedure (17). Broadly this comprises the following steps; see the code linked above and the referenced manuscript for details:

1. Background correction of expression values using negative control probes.
2. Transform the expression values by *x*_2_ = log_2_ (*x_raw_*)
3. Filter out genes expressed below detection threshold (*p* < 0.01).
4. Filter out rare genes expressed in fewer than 15% of our observations.
5. Map Illumina probe IDs to gene symbols.
6. Remove probes with low annotation quality.
7. If several probes map to the same gene, keep the one with the highest inter-quartile range in its measurements.
8. Define differential gene expression as the difference in log_2_ expression between a given case and its corresponding control.

Finally, as a data reduction step, we removed 2012 genes where the mean signal was more than 20 times the size of the standard deviation, i.e., genes that show little variation. After preprocessing, there were 6664 genes left in the gene expression matrix.

### Analysis Suggested by DAG

**Figure 1:**
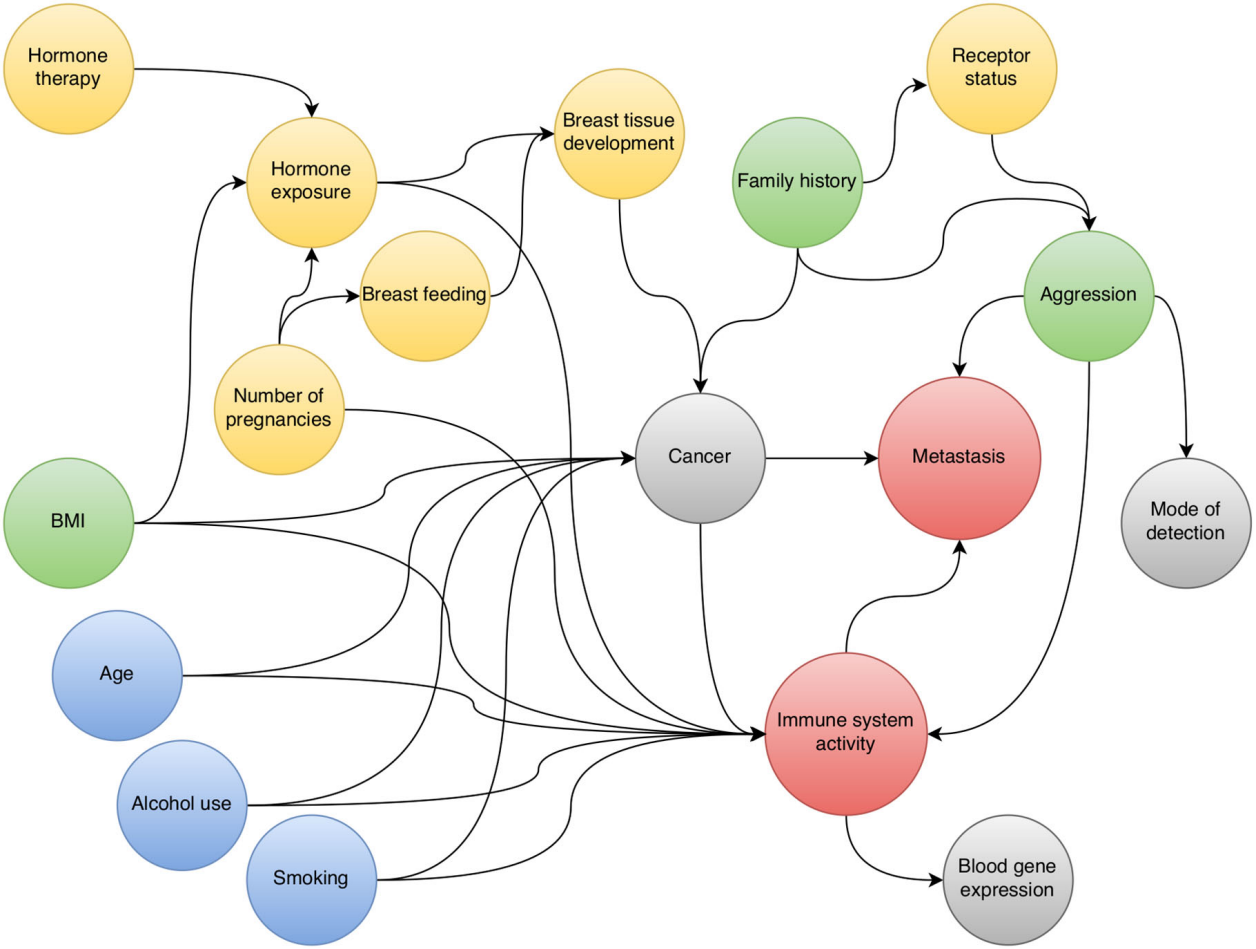
Directed acyclic graph for the relationship between pre-diagnostic blood gene expression and breast cancer metastasis.

We investigated the relationship between immune system activity, as measured by blood gene expression, and breast cancer metastasis. To guide this investigation we mapped out a causal diagram to the best of our ability. The diagram (**Figure 1**) suggests that we can obtain a causal estimate by adjusting for “aggression”. Since we cannot measure the aggressiveness of a cancer directly, we used detection mode as a proxy variable that could provide partial de-confounding. We hence estimated separate sets of parameters for screening cancers and interval cancers.

### Model

We modeled metastasis probability for a single gene *g*, *p_g_*, as a function of differential expression, *x_g_* (i.e., log_2_ *expression_case_* – log_2_ *expression_control_*). We did this by a Bayesian hierarchical model, stratifying by mode of detection, *s* ∈{screening, interval} with partial pooling between strata. For the observed metastatic status of person *i*, *y*_i_ ∈{0, 1}, we specified the following model:

(1) $$y_{i}∼\text{Bernoulli}\left(p_{g,i}\right),$$

(2) $$\text{logit}\left(p_{g,i}\right)=\alpha_{g,s_{i}}+\beta_{g,s_{i}}x_{g,i},$$

(3) $$\alpha_{g,s_{i}}∼\text{normal}\left(-1,\;1\right),$$

(4) $$\beta_{g,s_{i}}∼\text{normal}(\mu_{g,}\sigma_{g}),$$

(5) $$\mu_{g}∼\text{normal}\left(0,\;0.1\right),$$

(6) $$\sigma_{g}=e^{{\sigma^{\prime }}_{g}},$$

(7) $${\sigma^{\prime }}_{g}∼normal\left(0,\;0.2\right).$$

The function logit(*p*) = log(*p*) − log(1 − *p*) is the logarithmic odds of metastasis. Equations 1–4 describe a logistic regression with varying slopes between detection methods. I.e. we considered the association between risk and differential expression to be similar but not identical for screening and interval cancers. Equations 5–7 define the hyperpriors for the slopes. Equations 6, 7 imply a lognormal distribution for *σ_g_*. We used this non-centered parameterization because it makes the estimation faster and more reliable. We estimated these models using the NUTS sampler implemented in Stan (18). Having estimated the posterior distributions for our parameters in each detection setting, we integrated the detection setting out to obtain the average causal estimates.

$$\alpha_{g}∼\sum_{s}p(s)\alpha_{g,s},$$

$$\beta_{g}∼\sum_{s}p(s)\beta_{g,s}.$$

### Excess Risk

We standardized *χ_g_* to have mean zero and standard deviation of unity. This makes logit^−1^ (*α_g_*) the metastasis probability, or risk, for an individual with average differential expression of gene *g*. Likewise the quantity logit^−1^ (*α_g_* + *zβ_g_*) is the risk for someone with differential expression *z* standard deviations higher than the average. We call the quantity

$$\rho_{g}={\text{ logit}}^{-1}(\alpha_{g}+\;0.1\beta_{g})-{\text{ logit}}^{-1}(\alpha_{g})$$

the excess risk of metastasis for gene *g*. We chose *Z* = 0.1 because in our data most differences in means between metastases and non-metastases fall between ± 0.1 standard deviations. Hence we considered this a reasonable increase in differential expression for our investigation.

Excess risk is a signed quantity on the absolute scale. An excess risk of 0.01, or 1%, means that the risk of someone with elevated expression in a certain gene has a risk of 1% more than someone with average expression. I.e. if the risk associated with average expression is 25%, which it roughly tends to be, the risk of someone with 2% excess risk is 27%. A negative excess risk suggests that decreased expression has a higher metastatic risk, which implies under-expression among metastatic cases. We use excess risk throughout to assess how important the variation of a certain gene’s expression is for metastatic spread.

### Priors

**Figure 2:**
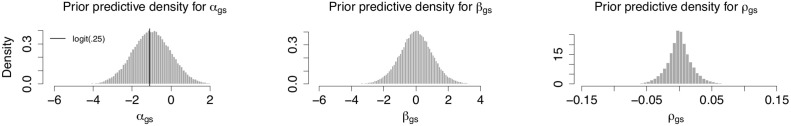
Prior predictive distributions. Prior predictive distributions for *α_gs_* and *β_gs_* along with the implied prior predictive distribution for excess risk of breast cancer metastasis.

We chose our priors to provide a slight shrinkage toward the null effect. The prior parameters are chosen *ad hoc* to provide a relaxed coverage of the parameter sizes we see fitting gene-wise maximum likelihood regressions for other outcomes (smoking and similar). This discourages outrageous estimates while still lending credence to realistic sizes. **Figure 2** shows prior predictive distributions for *α_gs_* and *β_gs_* along with the implied prior predictive distribution for excess risk. We have centered the prior distribution for *α_gs_* , the log odds for someone with average expression, on what roughly corresponds to a risk of 25%, which is what is seen in the population. The prior implied excess risk is sharply peaked around 0 and has the middle 60% of its mass in the range ± 0.014.

### Ranking Genes and Gene Sets

The **sign probability** of an excess risk is *p* (*ρ_g_* > 0) when the median of *ρ_g_* is positive and *p*(*ρ_g_* < 0) when the median is negative. This probability lies between.5 and 1 and expresses how much of the density for *ρ_g_* lies away from zero. A high sign probability means that we are quite sure of the direction of an excess risk but does not say anything about its magnitude. We used sign probability both to rank genes and to rank gene sets.

We ranked genes in decreasing order by sign probability and examined the first 100.

We ranked gene sets by the average sign probability in a given set. We extracted gene sets from the Molecular Signatures Database v.7.0 (MSigDB, (19)), using the following collections: Hallmark gene sets (H, (20)), Curated gene sets (C2), and Gene ontology gene sets-Biological processes (C5 BP). We examined the top 50 sets among these collections.

### Ethical Considerations

The NOWAC study was approved by the Norwegian Data Inspectorate and the Regional Ethical Committee of North Norway (reference: REK NORD 2010/2075). All women gave written, informed consent. Collection and storage of biological material was approved by the REK in accordance with the Norwegian Biobank Act (reference: P REK NORD 141/2008 Biobanken Kvinner og Kreft ref. 200804332-3).

## Results

In our study population of 394 middle-aged women (197 cases, 197 controls), age, BMI, smoking, and parity were similar between breast cancer cases and controls (**Table 1**). HT use among cases was slightly higher than among controls. Most cancers diagnosed in the mammography screening program were metastasis-free (82%, **Table 2**). This was much lower among those diagnosed in the interval (53%). This difference lends credibility to the decision to stratify by detection mode. The Luminal A, Triple negative, and HER2 positive subtypes were slightly more common among the metastasized cancers. However, for the latter two subtypes, the number of cases in each group is very small, so it is difficult to draw conclusions (**Table 2**).

**Table 1:** Descriptive characteristics of breast cancer cases and healthy controls.

	Controls	Breast cancer,non-metastasized	Breast cancer, metastasized
n	156	115	41
Age	56.1	56.1	56.2
BMI	25.5	25.6	26.2
Smoking	37 (24%)	26 (23%)	10 (24%)
HT use	29 (19%)	41 (36%)	12 (29%)
Parity	1.9	1.8	1.8

BMI, body mass index; HT, hormone therapy.

**Table 2:** Characteristics of the breast cancer cases.

	Non-metastasized	Metastasized
Follow-up time	319	376
Detection mode		
Screening	91 (79%)	20 (49%)
Interval	24 (21%)	21 (51%)
Subtypes		
Luminal A	59 (51%)	26 (63%)
Luminal B	9 (8%)	4 (10%)
Triple negative	2 (2%)	3 (7%)
HER2 positive	0	3 (7%)
Unknown	45 (39%)	5 (12%)

### Shrinkage Size

**Figure 3:**
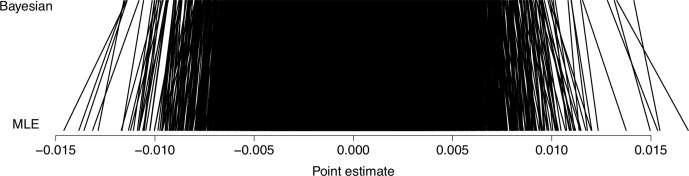
Shrinkage of predictions. Posterior mean predictions from our Bayesian models, compared to classical maximum likelihood estimates (MLE) in terms of predicted excess risk of breast cancer metastasis.

**Figure 3** shows a comparison of excess risk estimates between our posterior mean predictions and classical maximum likelihood estimates. As expected, there is a slight shrinkage toward an excess risk of null.

### Genes and Gene Sets Associated With Excess Risk of BC Metastasis

**Figure 4:**
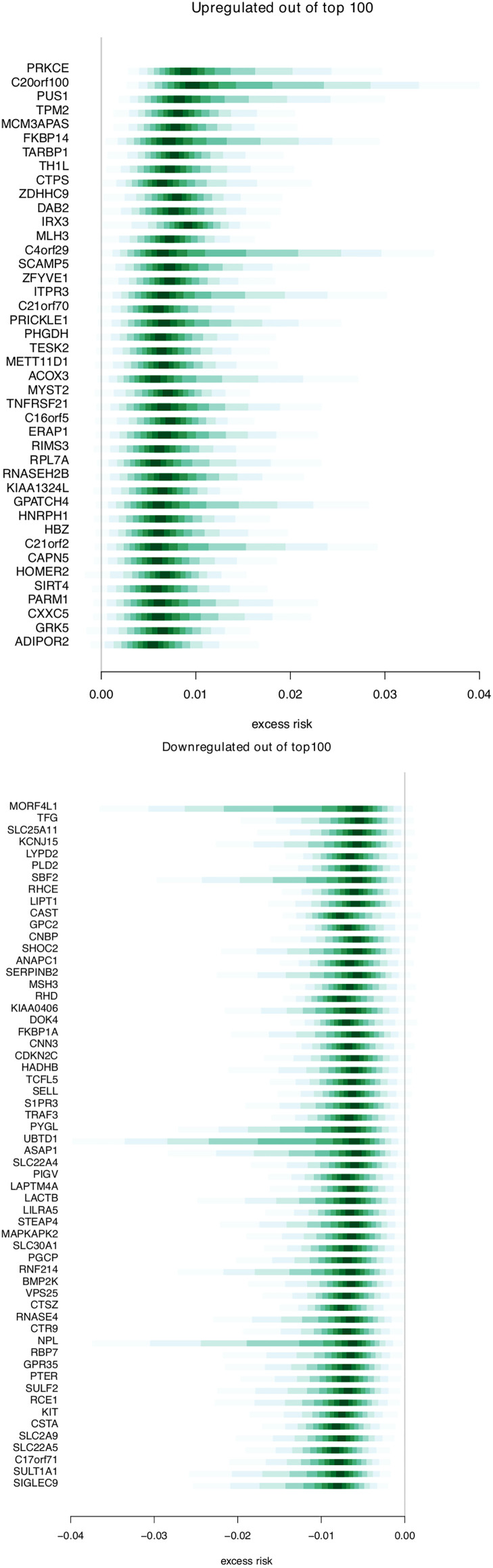
Genes associated with breast cancer risk. Distributions for excess risk of breast cancer metastasis for the up-regulated and down-regulated genes that were present among the top 100 genes associated with risk. The middle area shaded with the deepest value is the region between the 0.45–0.55 quantile. Each lightening of value extends these quantiles .05 in each direction (i.e. 0.4–0.6, 0.35– 0.65, etc.).

**Figure 4** shows the estimated excess risk of metastasis for the 100 genes with the highest sign probability. The first part shows up-regulated genes, and the second shows down-regulated genes. We show the full list of 100 genes in **Supplementary Table 1**. Out of these 100 genes, 42 were associated with increased risk (over-expressed in metastatic cases), and 58 were associated with decreased risk (under-expressed in metastatic cases). Among the genes associated with increased risk, some have been previously described in plasmacytiod dentritic cells (pDCs), including TARBP1, TNFRSF21, TPM2, DAB2, SCAMP5, and RIMS3. There are also three genes related to glutamine metabolism (SIRT4, PHGDH, CTPS1). Among the 58 single genes associated with increased metastasis risk there are some related to heme metabolism (e.g. BMP2K, RHC, RHD, SLC22A4, SLC30A1), transmembrane transport of ammonium (e.g. SLC22A4, -5, RHCE, RHD) and cations (those of ammonium transport, as well as FKBP1A, SLC2A9, STEAP4). There are several genes related to immunological processes (e.g. TRAF3, LILRA5, SIGLEC9).

**Figure 5:**
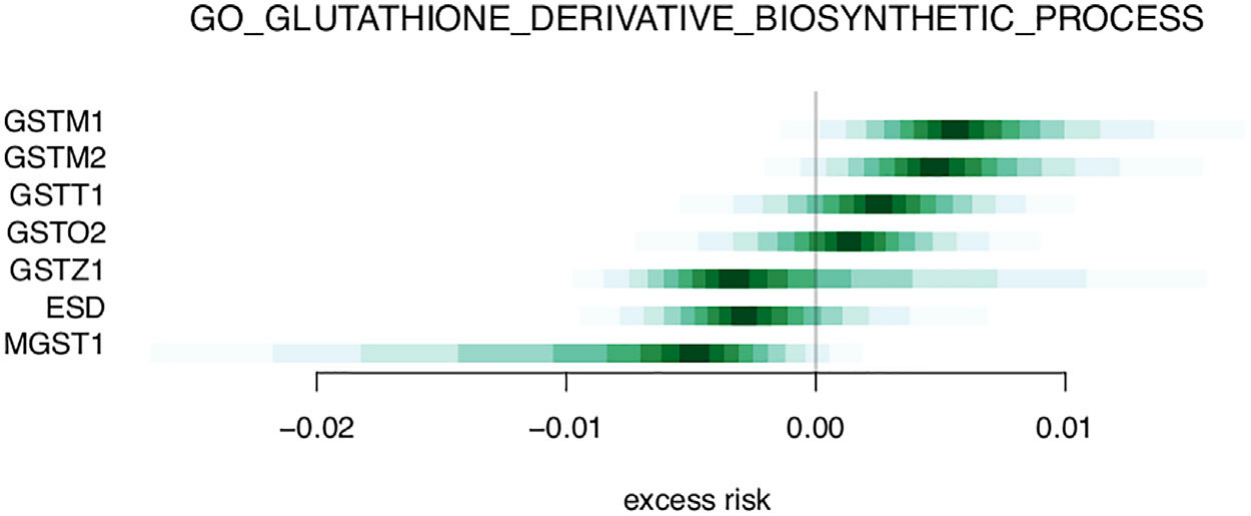
Excess risk estimates for genes of the GO_GLUTATHIONE_DERIVATIVE_BIOSYNTHETIC_PROCESS gene set.

**Figure 6:**
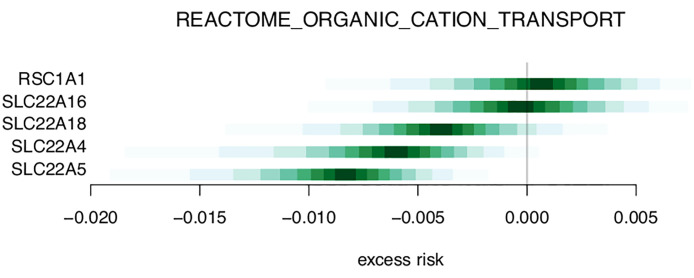
Excess risk estimates for genes of the REACTOME_ORGANIC_CATION_TRANSPORT gene set.

**Table 3** and **Supplementary Table 2** shows the top 20 and top 50 gene sets associated with risk of metastasis, respectively. The gene set results reflect the tendencies from the single gene analysis, with processes including glutathione derivative biosynthesis, ammonium transport, and immune functions (macrophage activation, IL2 signaling, antigen processing) being represented among the top 20 gene sets. In the gene set GO_GLUTATHIONE_DERIVATIVE_BIOSYNTHETIC_PROCESS (**Figure 5**), the seven included genes were associated with both increased risk (GSTM1, GSTM2) and decreased risk (MGST1). In contrast, four members of the SLC22 family were associated with decreased risk in the REACTOME_ORGANIC_CATION_TRANSPORT gene set (**Figure 6**). To identify the genes that drive the gene set results, we list genes present in multiple gene sets in **Table 4**. Their association with metastasis risk is displayed in **Supplementary Figure 1**. There were 18 genes present in three or more gene sets, four genes were present in four gene sets (GSTM1, GSTM2, SLC22A16, SLC22A4), and two genes were present in six gene sets (SLC22A5, SRC).

**Table 3:** Top 20 gene sets associated with risk of BC metastasis, ranked by the average sign probability.

Gene set name	Avg. sign p.
GO_AMMONIUM_TRANSMEMBRANE_TRANSPORT	0.866
GO_EPITHELIAL_CELL_CELL_ADHESION	0.853
GO_MACROPHAGE_ACTIVATION_INVOLVED_IN_IMMUNE_RESPONSE	0.839
REACTOME_INTERLEUKIN_2_SIGNALING	0.801
GO_SYNAPTIC_VESICLE_MATURATION	0.795
GO_NEGATIVE_REGULATION_OF_NEUROTRANSMITTER_SECRETION	0.789
GO_MITOCHONDRIAL_RNA_MODIFICATION	0.783
GO_GLUTATHIONE_DERIVATIVE_BIOSYNTHETIC_PROCESS	0.777
GO_PRIMARY_ALCOHOL_CATABOLIC_PROCESS	0.777
KEGG_DRUG_METABOLISM_CYTOCHROME_P450	0.777
GO_PHASIC_SMOOTH_MUSCLE_CONTRACTION	0.776
GO_NEGATIVE_REGULATION_OF_ACTIVIN_RECEPTOR_SIGNALING_PATHWAY	0.776
GO_ANTIGEN_PROCESSING_AND_PRESENTATION_OF_ENDOGENOUS_PEPTIDE_ANTIGEN	0.776
VALK_AML_CLUSTER_13	0.776
GO_POSITIVE_REGULATION_OF_CARDIAC_MUSCLE_CELL_DIFFERENTIATION	0.775
REACTOME_SYNTHESIS_OF_LEUKOTRIENES_LT_AND_EOXINS_EX	0.774
WENG_POR_TARGETS_GLOBAL_DN	0.769
GO_PEPTIDE_CATABOLIC_PROCESS	0.767
MATZUK_SPERMATOGONIA	0.767
GO_ENDODERMAL_CELL_FATE_COMMITMENT	0.767

Avg. sign p., average sign probability.

**Table 4:** Genes present in multiple gene sets.

gene	n	sets
SLC22A5	6	GO_AMMONIUM_TRANSMEMBRANE_TRANSPORT, GO_QUATERNARY_AMMONIUM_GROUP_TRANSPORT, REACTOME_IMPORT_OF_PALMITOYL_COA_INTO_THE_MITOCHONDRIAL_MATRIX, REACTOME_ORGANIC_CATION_ANION_ZWITTERION_TRANSPORT, REACTOME_ORGANIC_CATION_TRANSPORT, WENG_POR_TARGETS_GLOBAL_DN
SRC	6	GO_POSITIVE_REGULATION_OF_PODOSOME_ASSEMBLY, GO_TRANSCYTOSIS, PID_GLYPICAN_1PATHWAY, PID_NFKAPPAB_ATYPICAL_PATHWAY, REACTOME_EPHRIN_SIGNALING, REACTOME_P130CAS_LINKAGE_TO_MAPK_SIGNALING_FOR_INTEGRINS
GSTM1	4	GO_GLUTATHIONE_DERIVATIVE_BIOSYNTHETIC_PROCESS, GO_XENOBIOTIC_CATABOLIC_PROCESS, KEGG_DRUG_METABOLISM_CYTOCHROME_P450, KEGG_METABOLISM_OF_XENOBIOTICS_BY_CYTOCHROME_P450
GSTM2	4	GO_GLUTATHIONE_DERIVATIVE_BIOSYNTHETIC_PROCESS, GO_XENOBIOTIC_CATABOLIC_PROCESS, KEGG_DRUG_METABOLISM_CYTOCHROME_P450, KEGG_METABOLISM_OF_XENOBIOTICS_BY_CYTOCHROME_P450
SLC22A16	4	GO_AMMONIUM_TRANSMEMBRANE_TRANSPORT, GO_QUATERNARY_AMMONIUM_GROUP_TRANSPORT, REACTOME_ORGANIC_CATION_ANION_ZWITTERION_TRANSPORT, REACTOME_ORGANIC_CATION_TRANSPORT
SLC22A4	4	GO_AMMONIUM_TRANSMEMBRANE_TRANSPORT, GO_QUATERNARY_AMMONIUM_GROUP_TRANSPORT, REACTOME_ORGANIC_CATION_ANION_ZWITTERION_TRANSPORT, REACTOME_ORGANIC_CATION_TRANSPORT
ALDH3B1	3	GO_PRIMARY_ALCOHOL_CATABOLIC_PROCESS, KEGG_DRUG_METABOLISM_CYTOCHROME_P450, KEGG_METABOLISM_OF_XENOBIOTICS_BY_CYTOCHROME_P450
ARRB2	3	GO_POSITIVE_REGULATION_OF_CARDIAC_MUSCLE_CELL_DIFFERENTIATION, GO_POSITIVE_REGULATION_OF_CARDIOCYTE_DIFFERENTIATION, PID_NFKAPPAB_ATYPICAL_PATHWAY
EDN1	3	GO_PHASIC_SMOOTH_MUSCLE_CONTRACTION, GO_POSITIVE_REGULATION_OF_CARDIAC_MUSCLE_CELL_DIFFERENTIATION, GO_POSITIVE_REGULATION_OF_CARDIOCYTE_DIFFERENTIATION
EFNB2	3	GO_POSITIVE_REGULATION_OF_CARDIAC_MUSCLE_CELL_DIFFERENTIATION, GO_POSITIVE_REGULATION_OF_CARDIOCYTE_DIFFERENTIATION, REACTOME_EPHRIN_SIGNALING
GSTO2	3	GO_GLUTATHIONE_DERIVATIVE_BIOSYNTHETIC_PROCESS, KEGG_DRUG_METABOLISM_CYTOCHROME_P450, KEGG_METABOLISM_OF_XENOBIOTICS_BY_CYTOCHROME_P450
GSTT1	3	GO_GLUTATHIONE_DERIVATIVE_BIOSYNTHETIC_PROCESS, KEGG_DRUG_METABOLISM_CYTOCHROME_P450, KEGG_METABOLISM_OF_XENOBIOTICS_BY_CYTOCHROME_P450
GSTZ1	3	GO_GLUTATHIONE_DERIVATIVE_BIOSYNTHETIC_PROCESS, KEGG_DRUG_METABOLISM_CYTOCHROME_P450, KEGG_METABOLISM_OF_XENOBIOTICS_BY_CYTOCHROME_P450
KIT	3	GO_EPITHELIAL_CELL_CELL_ADHESION, GO_PHASIC_SMOOTH_MUSCLE_CONTRACTION, MATZUK_SPERMATOGONIA
LCK	3	PID_GLYPICAN_1PATHWAY, PID_NFKAPPAB_ATYPICAL_PATHWAY, REACTOME_INTERLEUKIN_2_SIGNALING
LTC4S	3	GO_LIPOXYGENASE_PATHWAY, REACTOME_SYNTHESIS_OF_LEUKOTRIENES_LT_AND_EOXINS_EX, RUAN_RESPONSE_TO_TNF_TROGLITAZONE_UP
MGST1	3	GO_GLUTATHIONE_DERIVATIVE_BIOSYNTHETIC_PROCESS, KEGG_DRUG_METABOLISM_CYTOCHROME_P450, KEGG_METABOLISM_OF_XENOBIOTICS_BY_CYTOCHROME_P450
SLC25A20	3	GO_AMMONIUM_TRANSMEMBRANE_TRANSPORT, GO_QUATERNARY_AMMONIUM_GROUP_TRANSPORT, REACTOME_IMPORT_OF_PALMITOYL_COA_INTO_THE_MITOCHONDRIAL_MATRIX

n, number of gene sets.

## Discussion

In this study we analyzed gene expression profiles in prospectively collected blood samples and examined genes and gene sets associated with risk of BC metastasis. Among the top genes, we identified pDC-related genes and processes like glutamine metabolism, several SLC22 transporters, and immune-related genes. Gene set analysis showed a similar overall picture. Among the up-regulated genes, estimates of excess risk lie mostly between 0 and.01, with some skew toward higher excess risk. The trend is similar among the down-regulated genes in the opposite direction. The sign probability for the top 100 genes was generally high, with no probability below 0.9. In the gene sets, average sign probability lay in the range.75–.85.

### Biological Aspects

Among the single genes associated with increased risk in our study, some have been previously described in plasmacytoid dendritic cells (pDCs) after vaccination against influenza (21). In general, pDCs are antigen presenting cells that initiate and coordinate immune responses. In line with these functions, antigen processing and presentation were among our top gene sets. pDCs have not been extensively studied in the cancer setting (22). Nevertheless, their presence in primary tumors were identified as negative prognostic markers for overall and relapse-free survival of breast cancer (23). In contrast, another study found positive association between circulating pDCs and breast cancer survival (24). In the latter study, there were lower levels of circulating pDCs in late stage cancers, but metastatic cancers were not investigated. The six genes related to pDCs were not found together in any of our investigated gene sets. This may be due to the fact that pDCs are somewhat newly described, and related gene sets may not be included in the MSigDB collections that we included for our gene set analysis.

Four members of the SLC22 family were among the genes most frequently found in our gene set data, all of which were associated with decreased risk of metastasis (i.e. down-regulated). Two were among the top 100 single genes associated with metastasis risk. This family of transporters are involved in diverse and ubiquitous processes like metabolism, and inter-organ and inter-organism signaling. A recent review suggests they have equal importance as the neuroendocrine system and growth factor-cytokine system (25). Their role in cation transport, carnitine handling, and drug/xenobiotic metabolism can be recognized in our results. However, as with all wide-ranging processes, specific hypotheses for the role of the SLC22 family in immune processes related to breast cancer metastasis cannot easily be reached based on transcriptomic data.

We identified three single genes of glutamine metabolism associated with increased risk of metastasis, and related gene sets were among our top identified gene sets. Glutamine is the body’s most abundant amino acid and is considered a “fuel for the immune system”. It is an essential amino acid for lymphocyte proliferation, cytokine production, and activities of macrophages and neutrophils (26). Low glutamine levels may impair immune cell function, with detrimental health effects (26). Based on our findings of differential expression of genes and gene sets related to glutathione, one might speculate that activity in the immune system is elevated in response to a metastatic tumor, as compared to that of a non-metastatic tumor.

### Methodological Aspects

DAGs are helpful for translating causal relationships into associations and create an overview of the subject matter as a basis for interdisciplinary discussions with the aim of designing an analysis strategy. But as with any method, there are strengths and weaknesses. Drawing the diagram to include all relevant assumptions is a challenge, also because absence of an arrow is a strong assumption. Nevertheless, drawing the DAG forces clarity about the underlying assumptions. To the extent that the diagram represents the true causal relationships, it helps identifying key sources of bias.

Our DAG is based on well-established risk factors for breast cancer. In the broad context, these risk factors act through two main mechanisms: either through DNA damage, or through hormone-related processes. Age, alcohol use, and smoking (**Figure 1**, blue) all increase cancer risk by causing accumulation of DNA damage in the cells. On the other hand, exposure to endogenous hormones (**Figure 1**, yellow) implies the exposure of cells to mitogenic substances. Higher hormone exposure levels (early onset of menarche, few or no pregnancies, use of HT), in combination with presence of non-maturated cells (late or no pregnancies, lack of breastfeeding) increases the risk of uncontrolled cell division in those cells. Responsiveness to hormone levels in both normal and cancerous cells depend on the presence of receptors like ER and PR. A few risk factors act through a combination of genetic and hormonal mechanisms (**Figure 1**, green). For example, in postmenopausal women, the fatty tissue produces estrogen (27); and excess body fat causes systemic inflammation and intracellular stress, which may increase DNA damage (28).

### Strengths and Limitations

Our data is from a case-control study nested within the prospective NOWAC Post-genome cohort. The advantage of this study design is that recall bias is reduced because exposure information is collected prospectively, before the onset of disease. Also, selection bias is reduced due to the prospective cohort being population-based. However, the nested case-control study cannot be used to infer causality between the exposure and the outcome. Specifically, it is not possible to measure and statistically control for all variables that may affect breast cancer metastasis. Hence the observed associations may be confounded.

The blood samples from all participants were taken before diagnosis of the disease. All the same, the cancer and/or the metastasis may already be present, but clinically undetected. We cannot determine if the gene expression profile is a cause or a consequence of the cancer and/or metastasis. This limitation also relates to the structure of the DAG: we have defined the gene expression profile, as a proxy for immune system activity, to be causally related to the metastasis. But this may not be biologically accurate. There is a very close and complex interaction between the immune system and the metastatic cancer that acts both *via* direct cell-cell contact and *via* excreted factors. Extracellular and intracellular signaling pathways are often redundant, two-way, and containing feedback loops. None of these mechanisms can be easily expressed *via* a DAG. One solution might be to map out the molecular two-way interactions and feedback loops as linear sequences of events in time (29), but this is beyond the scope of our work.

Although it is possible for us to build this DAG on the macro-scale of epidemiology, it is nigh impossible to do so on the molecular level. We have made no effort to do so and simply do gene-by-gene regressions. Hence there is almost certainly confounding on the molecular level, as genes are known to operate together in pathways.

Along with the causality-related limitations discussed above, which pertain to our study design and time of blood sampling, we stress that gene expression profiling is in its nature hypothesis generating. In line with this, we have chosen a statistical approach that focusses on model-based exploration as opposed to the testing of hypotheses. In building our statistical model we have made an effort to be scrupulous in reporting our assumptions. We made some of these choices for convenience, such as the use of single-gene regressions and the use of hard-coded prior parameters rather than a hierarchical model. We have explored other approaches to these data not reported here, notably (8, 30).

## Conclusion

In this work we have explored associations between breast cancer metastasis and prospective blood gene expression profiles. We conducted a Bayesian data analysis guided by a causal DAG to identify genes and pathways associated with risk of metastasis. Our results point to pDC function, the ubiquitous SLC22 family of transporters, and glutamine metabolism as candidates for future studies of the link between the immune system and metastasis.

We have identified potentially wide-reaching differences between metastatic and non-metastatic cases. The identified processes reflect both recently discovered links between the immune system and breast cancer metastasis, in the case of pDCs, and more well-described pathways, like regulation of the immune system by glutamine. Although the excess risk estimates are small in magnitude, our findings provide potentially important clues to the interaction between the immune system and metastasis.

## Data Availability Statement

The data analyzed in this study is subject to the following licenses/restrictions: Due to ethical restrictions on this dataset, which contains potentially sensitive patient information, the data will be made available upon request. Please contact the corresponding author (einar.j.holsbo@uit.no) and/or the host institution (info.helsefak@uit.no). Requests to access these datasets should be directed to Helsefak UiT (info.helsefak@uit.no); EH (einar.j.holsbo@uit.no).

## Ethics Statement

The NOWAC study was reviewed and approved by the Norwegian Data Inspectorate and the Regional Ethical Committee of North Norway (reference: REK NORD 2010/2075). All women gave written, informed consent. Collection and storage of biological material was approved by the REK in accordance with the Norwegian Biobank Act (reference: P REK NORD 141/2008 Biobanken Kvinner og Kreft ref. 200804332-3). The patients/participants provided their written informed consent to participate in this study.

## Author Contributions

Both authors designed the study. EH analyzed the data, and KO interpreted the results and wrote the manuscript. All authors contributed to the article and approved the submitted version.

## Funding

This study was funded by grants from UiT The Arctic University of Norway, and the European Research Council (ERC-AdG 232997-Tice).

## Disclaimer

Some of the data in this article are from the Cancer Registry of Norway. The Cancer Registry of Norway is not responsible for the analysis or interpretation of the data presented. The laboratory work and Illumina bead chip service was provided by the Genomics Core Facility (GCF), Norwegian University of Science and Technology (NTNU). GCF is funded by the Faculty of Medicine and Health Sciences at NTNU and Central Norway Regional Health Authority.

## Conflict of Interest

The authors declare that the research was conducted in the absence of any commercial or financial relationships that could be construed as a potential conflict of interest.
