# Supplementary figures and images for: Metastatic Breast Cancer and Pre-Diagnostic Blood Gene Expression Profiles—The Norwegian Women and Cancer (NOWAC) Post-Genome Cohort
Source: Front Oncol. 2020 Oct 15;10:575461. doi: 10.3389/fonc.2020.575461 (PMC7594625; doi:10.3389/fonc.2020.575461)

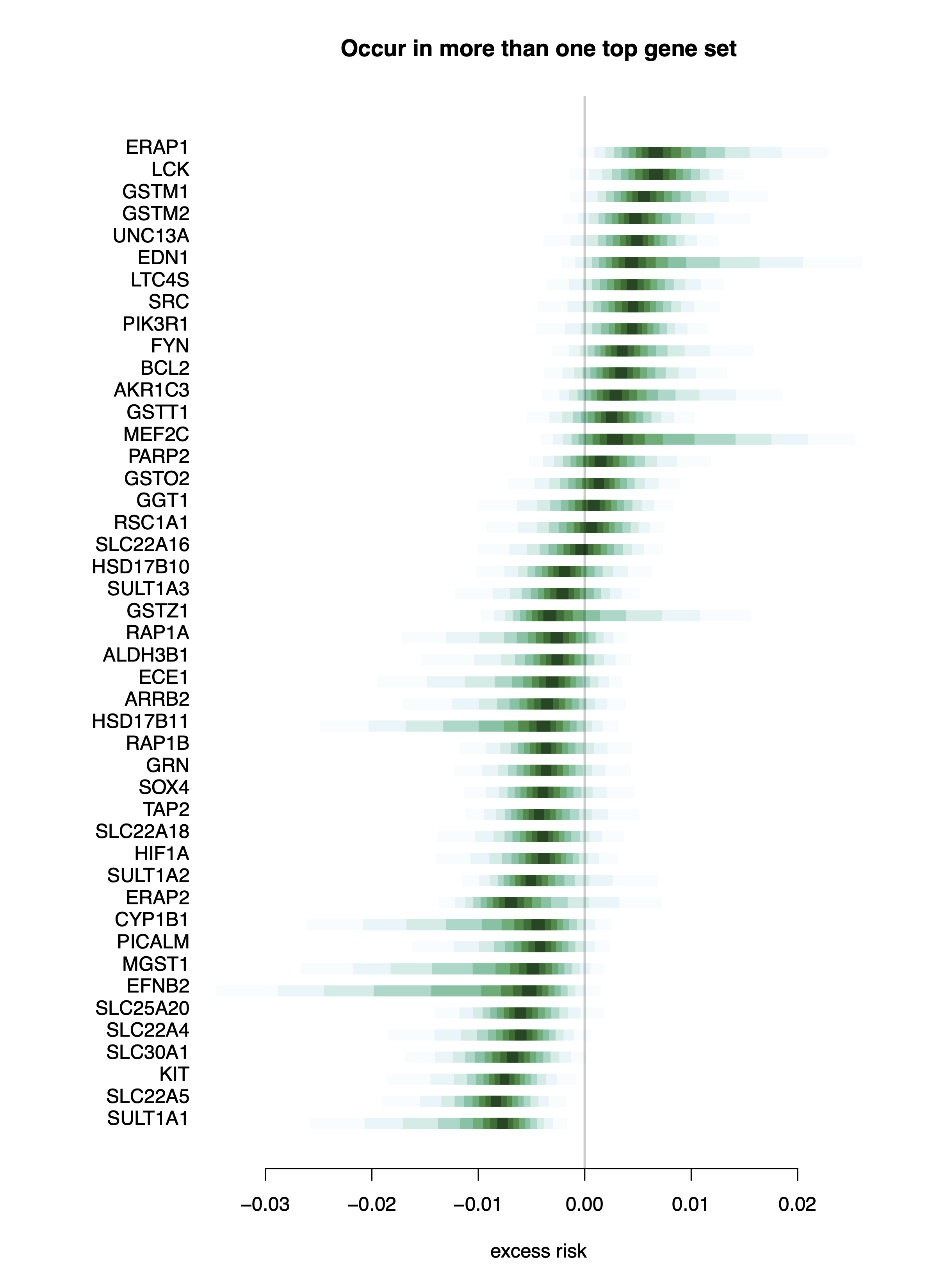

Supplement: Supplementary file 1 [file Image_1.png]
