# Supplementary material for: Metastatic Breast Cancer and Pre-Diagnostic Blood Gene Expression Profiles—The Norwegian Women and Cancer (NOWAC) Post-Genome Cohort
Source: Front Oncol. 2020 Oct 15;10:575461. doi: 10.3389/fonc.2020.575461 (PMC7594625; doi:10.3389/fonc.2020.575461)
Supplement: Supplementary file 2 [file Table_1.docx]

|  | **Average excess risk** | **Sign probability of excess risk** |
| --- | --- | --- |
| **PRKCE** | 0.0089 | 0.991 |
| **C20orf100** | 0.00966 | 0.991 |
| **PUS1** | 0.00823 | 0.982 |
| **SIGLEC9** | -0.00805 | 0.982 |
| **SULT1A1** | -0.0077 | 0.979 |
| **C17orf71** | -0.00785 | 0.977 |
| **SLC22A5** | -0.00831 | 0.975 |
| **TPM2** | 0.00811 | 0.974 |
| **SLC2A9** | -0.00755 | 0.973 |
| **MCM3APAS** | 0.00785 | 0.972 |
| **CSTA** | -0.00823 | 0.965 |
| **KIT** | -0.00751 | 0.963 |
| **FKBP14** | 0.00711 | 0.962 |
| **TARBP1** | 0.00775 | 0.961 |
| **RCE1** | -0.00731 | 0.961 |
| **TH1L** | 0.00724 | 0.96 |
| **SULF2** | -0.00679 | 0.96 |
| **PTER** | -0.0071 | 0.96 |
| **GPR35** | -0.00695 | 0.959 |
| **RBP7** | -0.00647 | 0.956 |
| **NPL** | -0.00622 | 0.954 |
| **CTR9** | -0.00693 | 0.954 |
| **RNASE4** | -0.00649 | 0.954 |
| **CTPS** | 0.00642 | 0.953 |
| **ZDHHC9** | 0.00797 | 0.953 |
| **DAB2** | 0.00763 | 0.952 |
| **IRX3** | 0.00918 | 0.951 |
| **CTSZ** | -0.00766 | 0.95 |
| **VPS25** | -0.00696 | 0.95 |
| **MLH3** | 0.0072 | 0.949 |
| **C4orf29** | 0.00652 | 0.949 |
| **BMP2K** | -0.00643 | 0.949 |
| **SCAMP5** | 0.0072 | 0.948 |
| **RNF214** | -0.00648 | 0.948 |
| **ZFYVE1** | 0.0072 | 0.945 |
| **PGCP** | -0.00687 | 0.945 |
| **SLC30A1** | -0.0068 | 0.944 |
| **MAPKAPK2** | -0.00636 | 0.944 |
| **STEAP4** | -0.00602 | 0.944 |
| **ITPR3** | 0.00659 | 0.94 |
| **PRICKLE1** | 0.00661 | 0.939 |
| **LILRA5** | -0.00657 | 0.939 |
| **C21orf70** | 0.00603 | 0.939 |
| **LACTB** | -0.00643 | 0.938 |
| **PHGDH** | 0.00629 | 0.938 |
| **TESK2** | 0.00639 | 0.938 |
| **LAPTM4A** | -0.00646 | 0.938 |
| **PIGV** | -0.00711 | 0.938 |
| **METT11D1** | 0.00659 | 0.937 |
| **ACOX3** | 0.00567 | 0.937 |
| **SLC22A4** | -0.00595 | 0.937 |
| **MYST2** | 0.00705 | 0.936 |
| **ASAP1** | -0.00579 | 0.936 |
| **TNFRSF21** | 0.00663 | 0.936 |
| **UBTD1** | -0.00599 | 0.936 |
| **C16orf5** | 0.00727 | 0.936 |
| **ERAP1** | 0.00667 | 0.935 |
| **PYGL** | -0.00651 | 0.935 |
| **TRAF3** | -0.00668 | 0.935 |
| **RIMS3** | 0.00615 | 0.933 |
| **RPL7A** | 0.00576 | 0.933 |
| **RNASEH2B** | 0.00677 | 0.932 |
| **KIAA1324L** | 0.00651 | 0.932 |
| **S1PR3** | -0.00596 | 0.93 |
| **GPATCH4** | 0.00604 | 0.93 |
| **SELL** | -0.00631 | 0.93 |
| **TCFL5** | -0.00634 | 0.93 |
| **HADHB** | -0.00599 | 0.93 |
| **HNRPH1** | 0.00625 | 0.929 |
| **HBZ** | 0.00602 | 0.929 |
| **CDKN2C** | -0.00596 | 0.929 |
| **CNN3** | -0.00674 | 0.929 |
| **FKBP1A** | -0.00583 | 0.929 |
| **C21orf2** | 0.00573 | 0.928 |
| **DOK4** | -0.00704 | 0.928 |
| **KIAA0406** | -0.00643 | 0.928 |
| **RHD** | -0.00756 | 0.927 |
| **MSH3** | -0.00658 | 0.927 |
| **SERPINB2** | -0.00568 | 0.927 |
| **ANAPC1** | -0.00658 | 0.926 |
| **CAPN5** | 0.00593 | 0.926 |
| **SHOC2** | -0.00563 | 0.926 |
| **CNBP** | -0.00576 | 0.925 |
| **HOMER2** | 0.00672 | 0.925 |
| **GPC2** | -0.00683 | 0.925 |
| **CAST** | -0.00776 | 0.925 |
| **LIPT1** | -0.00592 | 0.924 |
| **RHCE** | -0.00612 | 0.924 |
| **SIRT4** | 0.00582 | 0.924 |
| **PARM1** | 0.00615 | 0.923 |
| **SBF2** | -0.00571 | 0.921 |
| **CXXC5** | 0.00602 | 0.921 |
| **PLD2** | -0.00608 | 0.921 |
| **LYPD2** | -0.00656 | 0.92 |
| **KCNJ15** | -0.00566 | 0.92 |
| **GRK5** | 0.0065 | 0.919 |
| **ADIPOR2** | 0.00543 | 0.918 |
| **SLC25A11** | -0.00564 | 0.918 |
| **TFG** | -0.00541 | 0.918 |
| **MORF4L1** | -0.00562 | 0.918 |
