# Supplementary material for: Metastatic Breast Cancer and Pre-Diagnostic Blood Gene Expression Profiles—The Norwegian Women and Cancer (NOWAC) Post-Genome Cohort
Source: Front Oncol. 2020 Oct 15;10:575461. doi: 10.3389/fonc.2020.575461 (PMC7594625; doi:10.3389/fonc.2020.575461)
Supplement: Supplementary file 3 [file Table_2.docx]

| **Gene set** | **Average sign probability** |
| --- | --- |
| GO_AMMONIUM_TRANSMEMBRANE_TRANSPORT | 0.866 |
| GO_EPITHELIAL_CELL_CELL_ADHESION | 0.853 |
| GO_MACROPHAGE_ACTIVATION_INVOLVED_IN_IMMUNE_RESPONSE | 0.839 |
| REACTOME_INTERLEUKIN_2_SIGNALING | 0.801 |
| GO_SYNAPTIC_VESICLE_MATURATION | 0.795 |
| GO_NEGATIVE_REGULATION_OF_NEUROTRANSMITTER_SECRETION | 0.789 |
| GO_MITOCHONDRIAL_RNA_MODIFICATION | 0.783 |
| GO_GLUTATHIONE_DERIVATIVE_BIOSYNTHETIC_PROCESS | 0.777 |
| GO_PRIMARY_ALCOHOL_CATABOLIC_PROCESS | 0.777 |
| KEGG_DRUG_METABOLISM_CYTOCHROME_P450 | 0.777 |
| GO_PHASIC_SMOOTH_MUSCLE_CONTRACTION | 0.776 |
| GO_NEGATIVE_REGULATION_OF_ACTIVIN_RECEPTOR_SIGNALING_PATHWAY | 0.776 |
| GO_ANTIGEN_PROCESSING_AND_PRESENTATION_OF_ENDOGENOUS_PEPTIDE_ANTIGEN | 0.776 |
| VALK_AML_CLUSTER_13 | 0.776 |
| GO_POSITIVE_REGULATION_OF_CARDIAC_MUSCLE_CELL_DIFFERENTIATION | 0.775 |
| REACTOME_SYNTHESIS_OF_LEUKOTRIENES_LT_AND_EOXINS_EX | 0.774 |
| WENG_POR_TARGETS_GLOBAL_DN | 0.769 |
| GO_PEPTIDE_CATABOLIC_PROCESS | 0.767 |
| MATZUK_SPERMATOGONIA | 0.767 |
| GO_ENDODERMAL_CELL_FATE_COMMITMENT | 0.767 |
| GO_TRANSCYTOSIS | 0.766 |
| VANLOO_SP3_TARGETS_UP | 0.766 |
| GO_DENDRITE_EXTENSION | 0.766 |
| REACTOME_EPHRIN_SIGNALING | 0.765 |
| REACTOME_P130CAS_LINKAGE_TO_MAPK_SIGNALING_FOR_INTEGRINS | 0.765 |
| GO_HORMONE_CATABOLIC_PROCESS | 0.765 |
| REACTOME_ORGANIC_CATION_ANION_ZWITTERION_TRANSPORT | 0.765 |
| REACTOME_ORGANIC_CATION_TRANSPORT | 0.765 |
| GO_XENOBIOTIC_CATABOLIC_PROCESS | 0.764 |
| TORCHIA_TARGETS_OF_EWSR1_FLI1_FUSION_TOP20_DN | 0.764 |
| ROZANOV_MMP14_TARGETS_SUBSET | 0.764 |
| SCHRAETS_MLL_TARGETS_UP | 0.763 |
| GO_QUATERNARY_AMMONIUM_GROUP_TRANSPORT | 0.763 |
| GO_REGULATION_OF_TRANSCRIPTION_FROM_RNA_POLYMERASE_II_PROMOTER_IN_RESPONSE_TO_OXIDATIVE_STRESS | 0.763 |
| PID_GLYPICAN_1PATHWAY | 0.762 |
| KEGG_METABOLISM_OF_XENOBIOTICS_BY_CYTOCHROME_P450 | 0.762 |
| GO_POSITIVE_REGULATION_OF_PODOSOME_ASSEMBLY | 0.762 |
| KEGG_SULFUR_METABOLISM | 0.761 |
| GO_NEGATIVE_REGULATION_OF_INTERLEUKIN_6_SECRETION | 0.761 |
| CLAUS_PGR_POSITIVE_MENINGIOMA_DN | 0.76 |
| REACTOME_IMPORT_OF_PALMITOYL_COA_INTO_THE_MITOCHONDRIAL_MATRIX | 0.76 |
| GO_POSITIVE_REGULATION_OF_CARDIOCYTE_DIFFERENTIATION | 0.759 |
| GO_LIPOXYGENASE_PATHWAY | 0.759 |
| GO_CYTOPLASMIC_SEQUESTERING_OF_NF_KAPPAB | 0.758 |
| FUNG_IL2_SIGNALING_2 | 0.758 |
| DELACROIX_RAR_TARGETS_DN | 0.757 |
| RUAN_RESPONSE_TO_TNF_TROGLITAZONE_UP | 0.757 |
| GO_PYRIMIDINE_RIBONUCLEOSIDE_CATABOLIC_PROCESS | 0.757 |
| GO_POSITIVE_REGULATION_OF_CELL_MATURATION | 0.756 |
| PID_NFKAPPAB_ATYPICAL_PATHWAY | 0.756 |
